# Synthesis, Characterization and Application of 1-Butyl-3 Methylimidazolium Chloride as Green Material for Extractive Desulfurization of Liquid Fuel

**DOI:** 10.1155/2013/395274

**Published:** 2013-11-06

**Authors:** Swapnil A. Dharaskar, Mahesh N. Varma, Diwakar Z. Shende, Chang Kyoo Yoo, Kailas L. Wasewar

**Affiliations:** ^1^Advance Separation and Analytical Laboratory (ASAL), Department of Chemical Engineering, Visvesvaraya National Institute of Technology (VNIT), Nagpur, Maharashtra 440010, India; ^2^Environmental Management & Systems Engineering Lab (EMSEL), Department of Environmental Science and Engineering, College of Engineering, Kyung Hee University, 1 Seocheon-dong, Giheung-gu, Yongin-si, Gyeonggi-do 446-701, Republic of Korea

## Abstract

The possible application of imidazolium ionic liquids as energy-efficient green material for extractive deep desulfurization of liquid fuel has been investigated. 1-Butyl-3-methylimidazolium chloride [BMIM]Cl was synthesized by nucleophilic substitution reaction of n-methylimidazolium and 1-chlorobutane. Molecular structures of the ILs were confirmed by FTIR, ^1^H-NMR, and ^13^C-NMR. The thermal properties, conductivity, solubility, water content and viscosity analysis of [BMIM]Cl were carried out. The effects of reaction time, reaction temperature, sulfur compounds, and recycling of IL without regeneration on dibenzothiophene removal of liquid fuel were presented. In the extractive desulfurization process, the removal of dibenzothiophene in n-dodecane using [BMIM]Cl was 81% with mass ratio of 1 : 1, in 30 min at 30°C under the mild reaction conditions. Also, desulfurization of real fuels with IL and multistage extraction were studied. The results of this work might offer significant insights in the perceptive use of imidazoled ILs as energy-efficient green material for extractive deep desulfurization of liquid fuels as it can be reused without regeneration with considerable extraction efficiency.

## 1. Introduction

Sulfur-containing compounds in transportation fuels are converted to SO_*x*_ by combustion, which is a major source of acid rain or air pollution [1]. In the past decade, enormous awareness has been paid to deep desulfurization of transportation fuels due to the stringent policy on sulfur content in fuel (<10 ppm). For environmental protection purpose [2], many countries have mandated reduction of sulfur level in fuels down to 10 ppm and, with more stringent regulatory constraint, it is a trend to achieve little to no sulfur fuels in the next several years [3, 4]. 

In India, the present norms were decided by the Central Pollution Control Board; the current value of total sulfur in liquid fuels is limited to 350 ppm which has to be lowered down into the lowest possible extent. Presently, the removal of sulfur-containing compounds is carried out by a catalytic hydrodesulfurization (HDS) [5]. It requires severe conditions of high temperature and high H_2_ pressure to produce low-sulfur light oil [6]. The efficiency of HDS is limited to treat dibenzothiophene (DBT) and its derivatives, owing to their steric hindrance on the sulfur atom [7]. 

Several technologies such as extractive desulfurization, selective adsorption, catalytic oxidation, oxidative desulfurization, and biodesulfurization were proposed. In petroleum and hydrocarbon industries, various solvents such as ethers, amines, alcohols, and other volatile organic compounds have been used for the options like extraction, absorption, azeotropic distillation, and so forth [8]. Conventional solvents have their own limitations in terms of environmental issue, recycle ability, and so forth which can be overcome by the use of ionic liquids (ILs) as green solvents [9]. Among these, deep extractive desulfurization is an attractive technology, as it can be carried out at ambient temperature and pressure. A good extractant must have good extractive ability for sulfur compounds, free of contamination to the fuels, nontoxicity, environmental benignity, and stability for repetitive use. Thus, developing a new extractant is a key work for the extractive desulfurization (EDS) process. 

Over the past decade, ILs have fascinated much interest and regarded as an environmentally benign solvent. ILs have been studied for many possible applications for green chemical processes, such as an ionic conductive matrix and reaction solvent [10–14]. Based on the excellent performance, many research studies have been done on the effectiveness of ILs in some extraction processes, such as desulfurization and denitrogenation. Bösmann et al. reported for the first time the extractive performance of Lewis acid ILs in desulfurization [15]. Based on the above summarizations, we concluded that [BMIM]Cl with mass ratio 1 : 1 between chlorine anion and imidazole cation has not been investigated in detail for EDS of liquid fuels. 

ILs are organic salts with organic cations and inorganic or organic anions having melting points less than 100°C and negligible vapor pressure [16]. They also exhibit many unique advantages compared to conventional organic solvents, such as wide temperature range of application, high thermal stability, nonflammability, wide electrochemical window, high electrical conductivity, and highly favorable solubility of molecules [17]. 

 One of the most attractive features of these ILs is that they can be easily synthesized for any specific application either by careful selection of the cation or anion or both or by attaching new functional groups, such as amide, nitrile, amine, sulfonic acid, ether, alcohol, carboxylic, and thiols, to the structure to impart the desired properties [18, 19]. 

 In the present work, imidazolium-based 1-butyl-3-methylimidazolium chloride [BMIM]Cl was synthesized. FTIR, ^1^H-NMR, and ^13^C-NMR analyses were carried out for the characterization of IL. Also, the thermal properties, conductivity, solubility, and viscosity analysis were investigated. Synthesized [BMIM]Cl has been used as energy-efficient green material for the possible application in EDS of liquid fuel. 

## 2. Experimental

### 2.1. Materials and Reagents

IL used in the experiment was synthesized using analytical grade chemicals. The CAS number, source, and grades of the chemicals used are as follows: 1-methylimidazole (CAS 616-47-7, Acros 99%), 1-chlorobutane (CAS 109-69-3, Acros 99%), acetone (CAS 20003-L25, SDFCL 99.5%), ethyl acetate (CAS 20108-L25, SDFCL 99.5%), dibenzothiophene (CAS 132-65-0, Acros 98%), octane (CAS 111-65-9, Acros 99%), thiophene (CAS 110-02-1, Sigma Aldrich 99%), and 3-methylthiophene (CAS 616-44-4, Sigma Aldrich 98%). All the chemicals were used without any further treatment. Real fuels were purchased from Local Petroleum Pump House, Nagpur, Maharashtra, India. 

### 2.2. Synthesis of [BMIM]Cl

 [BMIM]Cl was prepared by the reaction of equimolar amounts of 1-methylimidazole and 1-chlorobutane at 60°C using rota mantle and under reflux for 48 h and then cooled to room temperature. Then, the product was washed twice with ethyl acetate. The remaining ethyl acetate was removed by heating at 60°C under vacuum for 2 h. The solution was filtered and the remaining resulting product was obtained [BMIM]Cl. 

### 2.3. Preparation of Model Liquid Fuel

Model liquid fuel was composed of DBT dissolved in octane. The S content of the model liquid fuel containing DBT in octane was 500 ppmw. With the same method, the S content of model liquid fuel containing thiophene (T), benzothiophene (BT), and 3-methylthiophene (3-MT) was 500 ppmw, respectively. Commercial gasoline and diesel with S content 186 and 385 ppmw, respectively, were used as real fuels.

### 2.4. Extractive Desulfurization of the Model Liquid Fuel

 The desulfurization experiments were conducted in a 100 mL two-necked flask by mixing the model liquid fuel (10 mL) and specific amount of IL with the mass ratio of model liquid fuel to IL 5 : 1, 3 : 1, and 5 : 1 at 30°C in a water bath for 30 min with vigorous stirring. On completion of the reaction, after separation, the upper phase (model liquid fuel) was withdrawn and analyzed to determine the concentration of sulfur in model liquid fuels. (%) removal of sulfur can be calculated by the following equation as

(1) (%)S removal=([DBT]Initial−[DBT]Final)[DBT]Initial×100.

### 2.5. Sulfur Content Analysis

 The model liquid fuel was analyzed by X-Ray Fluorescence Spectrometer (XRF), Model PW 2404, Phillips (now, PANAlytical, The Spectris Technology, Netherland), Centre of Sophisticated Analytical Instrumental Facility, Indian Institute of Technology (IIT), Mumbai, Maharashtra, India, to determine the concentration of DBT in the model fuels before and after desulfurization. 

### 2.6. Characterization of [BMIM]Cl

FTIR, ^1^H-NMR, and ^13^C-NMR analyses were carried out for characterization of IL. The thermal properties (TGA), conductivity, water content, solubility, and viscosity analysis of IL were also presented. The production of pure ILs is very important since impurities have a strong influence on their physical properties and stability. 

#### 2.6.1. FTIR Analysis

**Figure 1:**
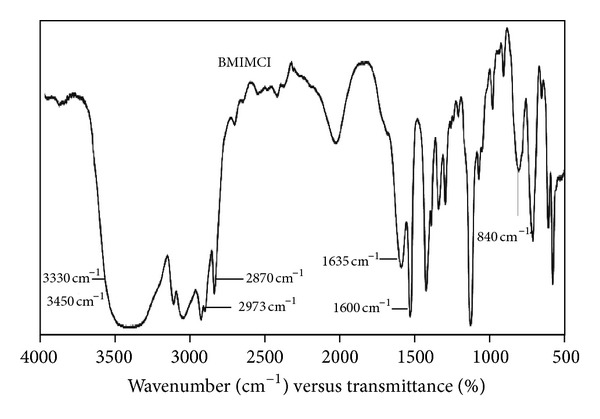
FTIR spectra of [BMIM]Cl.

A structure of the [BMIM]Cl was analyzed by Fourier transform infrared (FT-IR) Shimadzu IR-Affinity 1 Spectrometer (Japan), using the method of KBr pellet. The results were shown in Figure 1.

The peaks of wave numbers of 2973 cm^−1^ and 2870 cm^−1^ are the aliphatic asymmetric and symmetric (C–H) stretching vibration is due to methyl groups. A broad peak in the range 3330–3450 cm^−1^ is due to quaternary amine salt formation with chlorine. Wave numbers 1635 cm^−1^ and 1600 cm^−1^ are due to C=C and C=N stretchings. Peak at wave number 840 cm^−1^ is due to C–N stretching vibration. Based on FTIR analysis, it was observed that synthesized IL was [BMIM]Cl.

#### 2.6.2. ^1^H-NMR and ^13^C-NMR Analysis of [BMIM]Cl

 The [BMIM]Cl was characterized by ^1^H-NMR and ^13^C-NMR using CDCl_3_ as solvent on a spectrometer (Varian, USA Mercury plus 300 MHz for ^1^H-NMR and 76 MHz for ^13^C NMR spectrometer) for the determination of molecular structures and conformations. For all NMR analysis, approximately 30 mg of the IL was added into a 5 mm NMR tube. A stem coaxial capillary tube was loaded with 0.5 mL of solute. 

**Figure 2:**
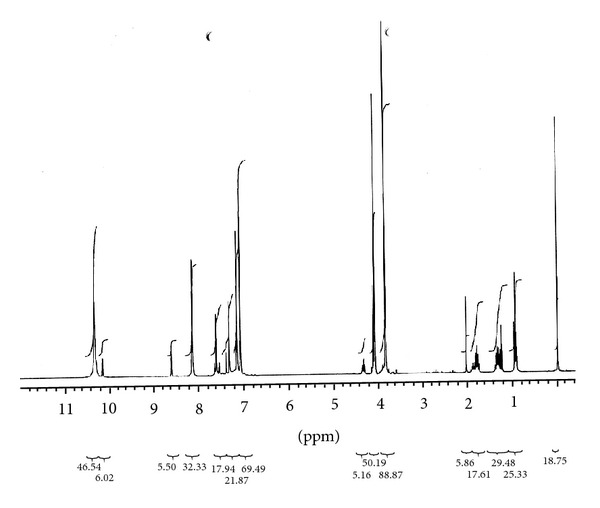
^1^H-NMR spectra of [BMIM]Cl.

**Figure 3:**
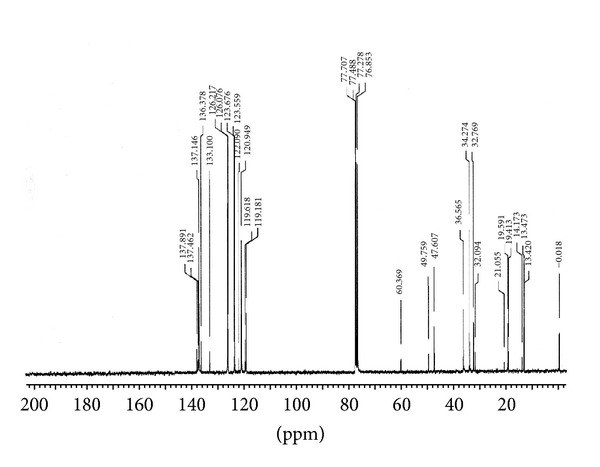
^13^C-NMR spectra of [BMIM]Cl.

^1^H-NMR data as shown in Figure 2 in ppm (*δ*) from the internal standard (TMS, 0.0 ppm), chemical shift (multiplicity, integration), and ^13^C-NMR data shown in Figure 3 in ppm (*δ*) from the internal standard (TMS, 0.0 ppm) were reported. The results of ^1^H-NMR and ^13^C-NMR analysis of [BMIM]Cl are given as follows:

  
^1^H-NMR  (300 MHz, CDCl_3_): *δ* (ppm) 7.147 (2, 1H, t), 3.839 (4, 2H, t,), 1.804 (5, 2H, tt), 1.348 (6, 2H, tq), 0.967 (7, 3H, t), 7.571 (8, 1H, dd), 7.198 (9, 1H, dd), 4.109 (10, 3H);
  
^13^C-NMR  (76 MHz, CDCl_3_): *δ* (ppm) 137.14 (C2), 126.2 (C4, d), 123.6 (C5, d), 47.60 (C6, d), 32.769 (C7), 19.59 (C8), 13.47 (C9), 34.27 (C10).

#### 2.6.3. Thermal Analysis

Thermal stability of [BMIM]Cl was determined with SII Co. Exstar TG/DTA (Japan) Thermo-gravimetric analyzer in order to know their upper temperature limit, and also melting point was measured with SII Co. Exstar AS-3 DSC-7020 (Japan) differential scanning calorimetry. 

**Figure 4:**
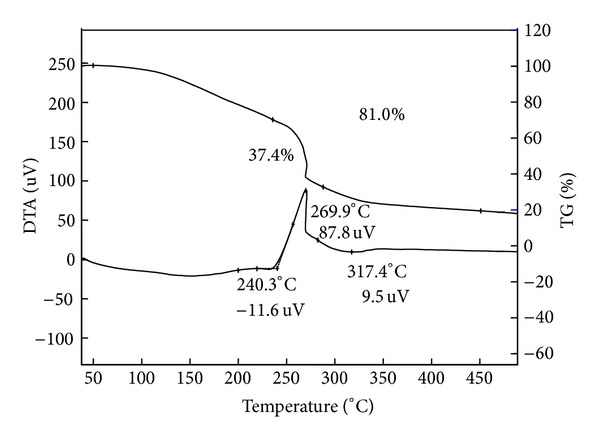
TGA analysis of [BMIM]Cl.

The sample (10–20 mg) was placed in an aluminum pan and heated over a temperature range of 30–500°C at a heating rate of 20°C min^−1^. The total time given to analyze the thermal stability was 23.5 min over temperature range from 30 to 500°C. The onset of thermal decomposition started at 240.3°C with the weight loss of 37.4% and decomposition ends around 317.4°C with weight loss of 81% as shown in Figure 4. No further decomposition was observed. This shows a high thermal stability of [BMIM]Cl.

**Figure 5:**
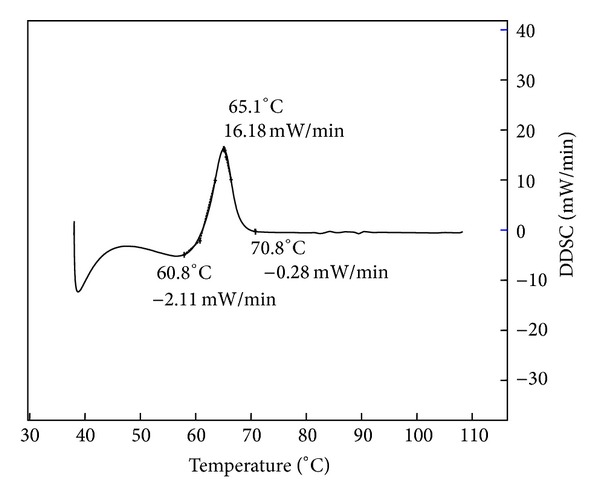
DSC analysis of [BMIM]Cl.

The melting points of synthesized ILs are the important indicator of an IL analyzed by differential scanning calorimetry (DSC). The melting point was measured from the highest position at the peak of the heating cycle. The melting point determined with DSC obtained only one transition, indicating that the product was pure. The melting point of [BMIM]Cl measured with DSC was 65.1°C and the results are shown in Figure 5. 

#### 2.6.4. Conductivity Analysis

Generally, ionic conductivity is proportional to the number of charge carrier ions and their mobility [20]. Conductivity of [BMIM]Cl was measured by PICO^+^ (Lab India) pH potentiometry/conductivity meter. [BMIM]Cl shows conductivity value of 460 (*μ*s·cm^−1^) which is relatively small. After anion exchanging, the conductivity could be increased. So, as compared to traditional organic solvents, IL has great advantages [21].

#### 2.6.5. Viscosity Analysis

**Figure 6:**
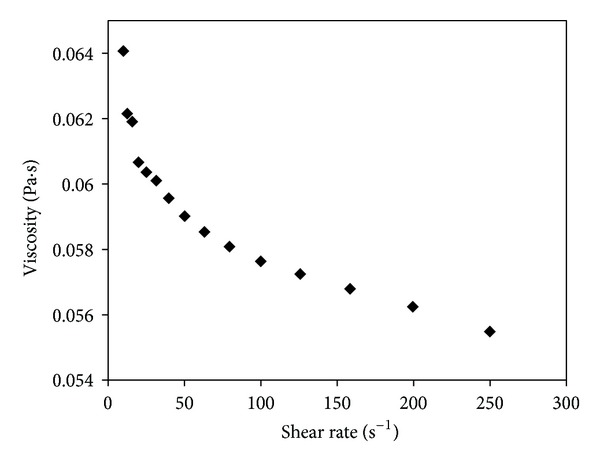
Effect of shear rate on viscosity of [BMIM]Cl.

 Viscosity of [BMIM]Cl was measured using ARG2 Rheometer (TA instruments, USA). Viscosity of IL varies inversely with respect to shear rate, it is well known that larger cations make IL more viscous because of the increased intermolecular van der Waals interactions [11, 13].  Figure 6 indicates that as shear rate increases from 0 to 250 (S^−1^), viscosity of [BMIM]Cl decrease from 0.064 to 0.055 (Pa·s), respectively. Also it was observed that optimum viscosity was found 0.055 at shear rate 250 (S^−1^). Similar results were reported in the literature [15].

**Figure 7:**
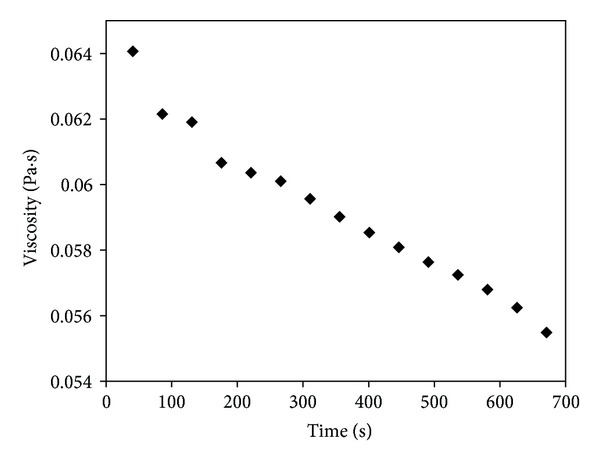
Effect of reaction time on viscosity of [BMIM]Cl at constant temperature (298.2 K).

Viscosity of [BMIM]Cl decreases with respect to reaction time at constant temperature (298.2 K), Figure 7; it was observed that the optimum value of viscosity was obtained at 671 S. Similar results were suggested in the literature [22].

#### 2.6.6. Water Content Analysis

Certain amount of water content was found more or less in all heavy oil reservoirs. It is key point whether water has effect on the viscosity reduction of heavy fuel by ILs. Water content of [BMIM]Cl was measured by MA-101-C Karl Fischer Titration System (Spectralab Instruments Pvt. Ltd.). The moisture content in [BMIM]Cl was found to be 6.85%. It has been reported that less than 10% of moisture content may enhance the extraction ability of IL [23]. 

#### 2.6.7. Solubility Analysis

 The solubility of [BMIM]Cl with six conventional solvents was studied. [BMIM]Cl may be dissolved in some conventional organic solvents such as methanol, acetonitrile, ethanol, acetone, and water but not all the organic solvents (e.g., [BMIM]Cl not dissolved in ethyl acetate). ILs solubility might be changed by changing the anions. Novel two-phase system can be created and used for various applications such as synthesis and extraction [21]. For the application of IL extractant, the solubility mechanism of IL is needed. Imidazolium-based ILs solubility in liquid fuel may give rise to extractant loss and liquid fuel contamination. This results suggest that the solubility of imidazolium-based IL in liquid fuel has to be optimized for future applications.

## 3. Results and Discussion

### 3.1. Effect of Reaction Time on S-Removal

The extractions of model fuel (DBT in octane) with [BMIM]Cl were carried out for 5, 10, 20, and 30 min at 30°C with mass ratios of 5 : 1, 3 : 1, and 1 : 1 (Mass ratio of model liquid fuel to IL). The desulfurization process went quiet quickly and sulfur concentration reduced from 500 to 160.2 ppmw (S-removal 68%), 134.3 ppmw (S-removal 73%), and 104.5 ppmw (S-removal 79%) with mass ratios of 5 : 1, 3 : 1, and 1 : 1, respectively in 20 min. Consequently, S-concentration decreased continuously with increased extraction time and reduced from 500 to 149.5 ppmw (S-removal 70%), 122.9 ppmw (S-removal 75.4%), and 94.8 ppmw (S-removal 81%) with mass ratios of 5 : 1, 3 : 1, and 1 : 1, respectively, in 30 min. This is because, at the beginning of the reaction, there is high DBT content in the model fuel, the extraction rate becomes high, and S-removal rate increase quickly. 

**Figure 8:**
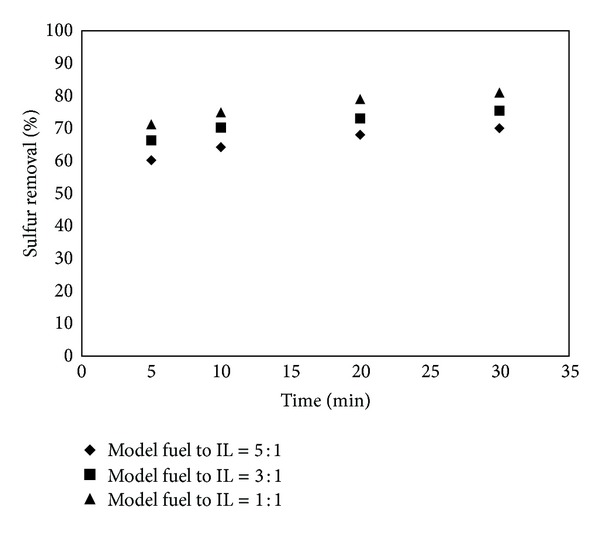
Removal of sulfur from model fuel (DBT in octane) using [BMIM]Cl at different extraction times (temperature = 30°C, extraction time = 5–30 min, initial Sulfur Concentration = 500 ppmw).

When the reaction has proceeded to certain extent, a small amount of DBT exists in the model fuel phase so that extraction rate becomes low and S-removal rate no longer markedly increases. The results, in Figure 8, show that 30 min of contact between the model liquid fuel and IL phase is more than sufficient to reach the equilibrium. So the optimum time required for the desulfurization of model fuel is 30 min. 

Imidazolium ILs are more capable of effectively extracting DBT than other S-containing compounds [24, 25]. This observation was also reported in other ILs extraction process for imidazolium ILs has been attributed to the higher polarisable electron *π*-electron density of DBT which tends to insert the molecular structure of ILs [26, 27]. Therefore, the better DBT removal ability of [BMIM]Cl expected to be recognized to enhance *π*-*π* interactions, because moreover the cations, the (–C*≡*N) group in the [BMIM]Cl anions may also interact with the thiophenic ring of DBT through *π*-*π* interaction [28, 29]. Consequently, [BMIM]Cl demonstrated the best DBT removal ability and was selected to investigate the effect of temperature, desulfurization of real fuel, multiple extraction, and ILs regeneration on extraction of S-removal.

### 3.2. Effect of Reaction Temperature on S-Removal

**Figure 9:**
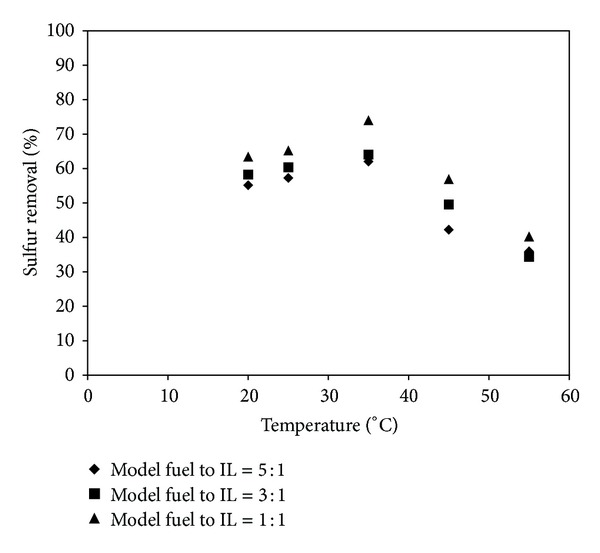
Removal of sulfur from model fuel (DBT in octane) using [BMIM]Cl at different extraction temperatures (°C) (temperature = 20−55°C, extraction time = 30 min., initial sulfur concentration = 500 ppmw).

Reaction temperature plays a key role in the EDS process; Figure 9, shows the effect of the reaction temperature (20°C, 25°C, 35°C, 45°C, 55°C) on S-removal. As shown in Figure 9, as the reaction temperature increased from 20°C to 35°C, the S-removal efficiency increases and then decreased. This effect may be attributed to that when the reaction temperature is below 45°C, the viscosity of IL is reduced with the increasing temperature, then the flexibility of IL is enhanced and the viscous flow layer is formed. Thus, the extraction efficiency of DBT in the model fuel by IL and the S-removal rate increase. When the reaction temperature exceeds 35°C, the flexibility of IL is not clearly enhanced. Moreover, the rate of S-removal will no longer increases and even to some extent declines [30]. Therefore, the best ability of [BMIM]Cl to extract DBT was reflected at 35°C which is taken as the optimal reaction temperature. The sulfur content of the model fuel decreased from 500 to 190.6 ppmw (S-removal 62%), 178.8 ppmw (S-removal 64%), and 129 ppmw (S-removal 74%) with mass ratios of 5 : 1, 3 : 1, and 1 : 1, respectively, as shown in Figure 9. 

There was remarkable drop in S-removal when the temperature reached 55°C, and the desulfurization was only 35.9%, 34.3%, and 40.2% with mass ratios of 5 : 1, 3 : 1, and 1 : 1, respectively. Insensitivity to temperature was also observed in other extraction systems such as [BPy]BF_4_ [31], [(CH_2_)_4_SO_3_HMIM]Tos [32], [BMIM]BF_4_, and [BMIM]PF_6_ [33]. Subsequently, sulfur extraction may be performed at room temperature, which is encouraging for less energy consumption.

### 3.3. Effect of Sulfur Compound on S-Removal

Table 1 shows the effect of the selected sulfur compounds typically found in diesel fuel and gasoline on extraction with pure hydrocarbons and [BMIM]Cl as a model extraction system. It can be seen that results for DBT, T, BT, and 3-MT are good. This indicated that the most likely mechanisms for the extraction of sulfur compounds with [BMIM]Cl are the formation of liquid clathrates and *π*-*π* interactions between aromatic structures of the extraction target and the imidazolium ring system [31]. Many nitrogen, oxygen, and aromatic compounds existed in the real fuel, which decreased the extraction performance of the IL for S-containing compounds.

 There were different kinds of alkyl-substituted DBTs in the actual diesel, such as BT, T, and 3-MT. The reports suggested that methyl-substituted DBTs are more reactive than DBT during oxidation reactions [34, 35]. However, the reactivity of the DBT decreased with increasing methyl substitutes at the derivative substitute positions; the reactivity sequencing was DBT > BT > T > 3-MT. 

### 3.4. Recycling of [BMIM]Cl without Regeneration

In practical processes, regeneration of ILs is important considering the high cost of ILs. The sulfur extraction performance of the [BMIM]Cl without regeneration is investigated and the results are shown in Table 2. The Spent [BMIM]Cl was reused upto four cycle for the possible extraction of sulphur removal. It was observed that the spent [BMIM]Cl was able to extract DBT from model liquid fuel even without regeneration, however, at a lower efficiency of 49.2% to 75.4% with spent [BMIM]Cl. This reduced S-removal might be attributed to DBT which dissolved in IL and decreased the extraction performance of IL [36]. This result indicated that after the [BMIM]Cl is recycled four times, the S-removal rate decreases slightly. 

From the aspect of industrial application, the S-content of fuels in a practical desulfurization process varies a great deal. Thus, an ideal IL extractant should have enough extraction capacity to assure the desulfurization performance. The maximum absorption capacity for DBT per mole is high enough for the practical desulfurization process (sulfur content is typically lower than 500 ppmw). The other important key factors are selectivity, partition coefficient, and regeneration ability which are constant irrespective of the sulfur content in fuels. Hence, imidazolium-synthesized ILs extraction performance (capacity, partition coefficient, selectivity) would not be affected by various conditions of real fuels [36].

### 3.5. Desulfurization of Real Fuels Using [BMIM]Cl

 The extraction of real fuels such as diesel and gasoline is much more complicated due to its complex chemical composition, including many different sulfur compounds and other impurities like organic nitrogen and oxygen compounds. The results of extractive desulfurization with real fuels are also promising. [BMIM]Cl display high S-removal capability from diesel and gasoline in single-stage extraction in 30 min at 30°C with mass ratios of 5 : 1, 3 : 1, and 1 : 1 in Table 3.

[BMIM]Cl exhibits the best sulfur extraction ability for S-removal in diesel which was reduced from initial sulfur of 385 to 210.4 pppw (45.3% S-removal), 160 ppmw (58.4% S-removal), and 132.1 ppmw (66% S-removal) with mass ratios of 5 : 1, 3 : 1, and 1 : 1 in single-stage extraction, respectively, as shown in Table 3. In gasoline, it was reduced from initial sulfur of 186 to 70 ppmw (61% S-removal), 54.8 ppmw (70% S-removal), and 46.8 ppmw (74% S-removal) with mass ratios of 5 : 1, 3 : 1, and 1 : 1 in single-stage extraction, respectively. 

Diesel and gasoline contain more complex compounds than the model fuel, such as nitrogen- and sulfur-containing compounds (alkylthiophene, benzothiophene) which decrease the ability of [BMIM]Cl for S-removal. Because of steric effect of alkyl group in the aromatic rings, methyl-thiophene, methyl-benzothiophene, methyl-dibenzothiophene, and so forth, sulfur-containing compounds in diesel and gasoline are extracted less than DBT in the model fuel by ILs [30].

### 3.6. Multistage Extraction Process

**Figure 10:**
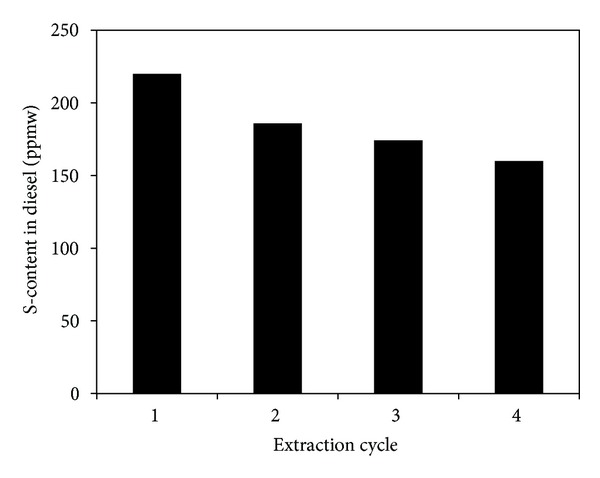
The multistage extraction performance of [BMIM]Cl for diesel (temperature = 30°C, mass ratio of diesel/IL = 5 : 1, extraction time = 30 min, initial sulfur concentration = 385 ppmw).

**Figure 11:**
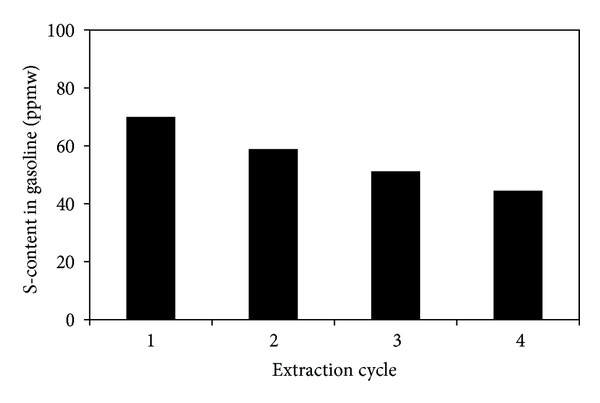
The multistage extraction performance of [BMIM]Cl for gasoline. (Temperature = 30°C, Mass ratio of gasoline/IL = 5 : 1, Extraction time = 30 min, Initial Sulfur Concentration = 186 ppmw).

Although a high S-removal by [BMIM]Cl is obtained as shown in Table 3 in a single-stage extraction, the final sulfur content in raffinate fuels cannot meet the specific requirement of low sulfur fuels (e.g., <50 ppm). Hence, multistage extractions are performed, and the results are shown in Figures 10 and 11. The sulfur content in diesel drops significantly from 385 to 160 ppmw after 4 cycles and the sulfur content in gasoline is reduced from 186 to 44.5 ppmw after 4 cycles. As a result, multiple extractions are effective to reduce the sulfur content of liquid fuel to considerably a negligible amount. Similar results were reported in the literature [36].

## 4. Conclusion

Imidazolium-based [BMIM]Cl can be used as an energy-efficient green material for the extractive deep desulfurization of liquid fuels, mainly with regards to those sulfur compounds that are very complex to remove by common hydrodesulfurization technique. [BMIM]Cl was the most efficient in the removal of DBT-containing liquid fuels. [BMIM]Cl is an economical, easily synthesized, and an excellent green material for sulfur-containing compounds. Thus, EDS method could be developed into a simple, mild, and environmentally benign method for deep desulfurization.

## 5. Highlights

- Imidazolium-based ILs were synthesized and employed as extractants for S-removal.
- [BMIM]Cl not only acts as extractant but also acts as a catalyst in the process of desulfurization.
- DBT-containing model fuel in [BMIM]Cl could reach 81% of S-removal with mass ratio 1 : 1 at 30°C in 30 min, which was the remarkable enhancement of EDS process over HDS.
- The IL could be reused without regeneration more than five times with a slight decrease in activity.
- The EDS process could be an option for environmentally benign method for deep desulfurization.

## Figures and Tables

**Table 1:** Effect of sulfur compounds on extraction with [BMIM]Cl.

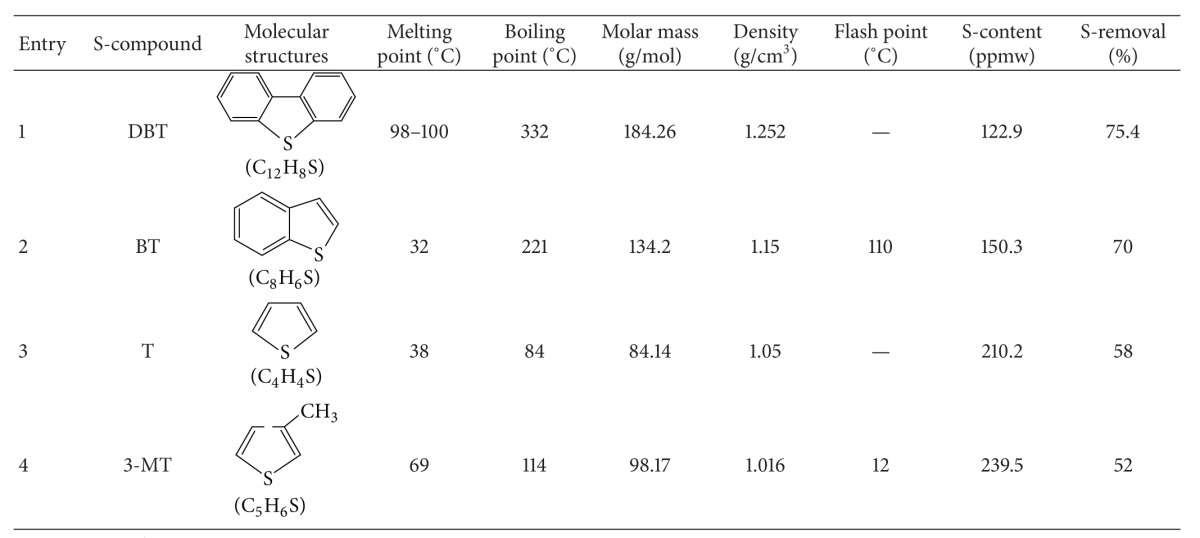

Temperature= 30°C, mass ratio of model fuel to IL = 3 : 1, extraction time = 30 min.

Initial sulfur concentration = 500 ppmw.

**Table 2:** Recycling of spent [BMIM]Cl without regeneration.

No. of cycle	Model fuel	S-content (ppmw)	S-removal (%)
1	(octane + DBT)	122.95	75.41
2		165.05	66.99
3		221.05	55.79
4		253.85	49.23

Temperature = 30°C, mass ratio of model fuel/IL = 3 : 1, extraction time = 30 min. Initial sulfur concentration = 500 ppmw.

**Table 3:** Desulfurization of real fuels with [BMIM]Cl.

Diesel to IL (mass ratio)	S-content (ppmw)	S-removal (%)	Gasoline to IL (mass ratio)	S-content (ppmw)	S-removal (%)
5 : 1	210.4	45.3	5 : 1	70.3	61
3 : 1	160	58.4	3 : 1	54.8	70
1 : 1	132.1	66	1 : 1	46.8	74

Temperature = 30°C, extraction time = 30 min, initial sulfur concentration of diesel and gasoline = 385 and 186 ppmw, respectively.

## Nomenclature

[BMIM]Cl: 1-Butyl-3-methylimidazolium chloride
IL: Ionic liquids
ppmw: Parts per million weight
DBT: Dibenzothiophene
T: Thiophene
BT: Benzothiophene
3-MT: 3-Methylthiophene
EDS: Extractive desulfurization system.
